# Green Jobs: Bibliometric Review

**DOI:** 10.3390/ijerph20042886

**Published:** 2023-02-07

**Authors:** Łukasz Jarosław Kozar, Adam Sulich

**Affiliations:** 1 Department of Labour and Social Policy, Faculty of Economics and Sociology, University of Lodz, ul. Rewolucji 1905 r. No 37, 90-214 Lodz, Poland; 2 Department of Advanced Research in Management, Faculty of Business Management, Wroclaw University of Economics and Business, ul. Komandorska 118/120, 53-345 Wroclaw, Poland; 3 Schulich School of Business, York University, 4700 Keele Street, Toronto, ON M3J 1P3, Canada

**Keywords:** green jobs, green labor market, green economy, sustainable development

## Abstract

Among the visible effects as Sustainable Development (SD) transitions from theory into practice, there are Green Jobs (GJs). There are multiple variants in naming this phenomenon in the labor market. Among them are green collars, green employment, and sustainable employment, all indicating a profound inconsistency in the GJ definition. This article aims to identify keyword-specified areas around which the topic of GJs revolves in the scientific literature indexed in the Scopus database. The usage of two methods has achieved this goal. First is the Structured Literature Review (SLR) variation with queries, and it is used to explore the scientific database to determine GJ’s definition consistency by the queries syntax. The second method is the search results analysis performed in the Scopus database online to identify the most cited publications and most contributing authors. Then the bibliometric analysis was performed to create bibliometric maps of the most critical keywords in VOSviewer software. The combination of those two approaches allowed this research to indicate the most influential research directions on GJs. The results are presented in graphical forms, and tables with main co-occurring keyword clusters were identified. GJs are a key part of green economy development, where green self-employment and green entrepreneurship play a pivotal role. The presented results can inspire other researchers who are looking for a research gap or describing the state of the art. Politicians and decision-makers can be influenced by the presented contextualization of green job’s meaning in the labor market.

## 1. Introduction

The technological transition from a brown economy (based mainly on the exploitation of fossil fuels) to a green economy (based on renewable energy sources) is a multidimensional, worldwide process [1,2] that involves all sectors of the modern economy [3,4]. This process is the result of the need to implement the idea of Sustainable Development (SD) in socio-economic practice [5,6]. The assumption is that the green economy is about conducting all economic activities with respect for nature’s assets and in such a way as to prevent irreversible changes to the environment [7]. Therefore, there are changes taking place in all business processes and decision-making aiming at a low-carbon, and a resource-efficient economy based on green technologies [8,9]. The examples of the changes towards SD are visible in all economic sectors and there is a gradual green transformation of the whole economy taking place [10,11]. This shift towards a green economy is particularly visible in the labor market [12,13]. In this economic area, there is the creation of new jobs or the change of existing jobs for the needs of a gradually greening or circular economy [14,15]. Such jobs created as a result of pro-environmental transformations of activities undertaken by various types of entities are called green jobs (GJs) [9,16,17]. GJs are the result of the ongoing transformation focused on SD [18] and are an important part of the scientific analyses raised around the topic of a green economy [19,20]. In particular, the scientific literature emphasizes that employees employed in GJs should be characterized by appropriate levels of knowledge [21,22], skills [23,24], attitudes [25], and pro-environmental behavior [26,27]. Hence, there are a growing number of scientific studies addressing the issue of the green competence of employees [28,29]. Based on previous research on the issue of GJs, it is possible not only to point out how they differ from non-green jobs [30,31], but also to draw the conclusion that it is on the degree of development of human capital in the context of sustainability issues that progress aimed at greening modern economies depends [32]. GJs can be characterized by both quality and degree of greening [9,33]. Thus, GJs can be called a kind of litmus test of the greening of modern economies of both the countries concerned and international communities [34].

There is an ongoing scientific discussion on the question of defining what GJs are [9,15]. This discussion is triggered by diverse scientific and research approaches to such specific positions [35,36]. The result of the considerations undertaken by researchers is the identification of GJs with given economic sectors [37,38], which are called green sectors [39,40], or with specific jobs [41,42]. According to the most popular definition of GJs, they represent “work in agricultural, manufacturing, research and development, administrative, and service activities that contribute substantially to preserving or restoring environmental quality” [43]. Recently, special attention has been paid to the energy sector [44,45], where it is indicated that the transformation of this sector towards renewable energy sources contributes to the creation of GJs [4,33]. In addition, researchers on the subject recognize another important aspect resulting from the indicated transformation, namely that the renewable energy sector creates more jobs per unit of energy than the fossil fuel-based sector [46,47]. Thus, what is occurring in this area is not only a qualitative change in jobs but also the creation of new GJs [48,49]. In light of the research conducted to date on the topic of GJs, a view directed toward qualitative research of all jobs in the economy in terms of their greening is also evident [9,50].

The aim of this article is to identify key areas around which the topic of GJs revolves in the literature. Such an area-based systematization is important since a significant systematic research gap is still apparent in this field. Previous literature studies aimed at this goal primarily ignore the issue of the different nomenclature of GJs [51], or analyze the indicated issues in rather narrow periods [52], and thus make the undertaken deductions incomplete. The research problems observed are the main reason for this study. The purpose of the article presented above is accompanied by verification of the statement that GJs and their nomenclature are constantly evolving, which in itself is an important field for researchers and management theory development.

To achieve a such goal to identify key areas and keywords, around which GJs are developed in the scientific literature, the bibliometric analysis was performed. The adopted method was Structured Literature Review (SLR) variation method with queries. The scientific database explored in this bibliometric study is the Scopus collection of peer-reviewed articles, conference proceedings, book chapters, etc. In this study, the whole collection was analyzed according to the presented in the Materials and Methods section methodology. Using the VOSviewer software (version 1.6.18) the co-occurrences networks were generated to identify associations between frequently occurring keywords in scientific articles indexed in Scopus.

Following the logic adopted in this paper as a bibliometric review, the presentation of the GJs subject is divided into four interrelated sections. After presenting in the first section the reasons for bibliometric research and explaining the purpose of the work, a description of the research methodology is presented in the second section. The results of the research are presented in the third section with the support of bibliometric maps and their descriptions. The article concludes with a discussion of the results, along with recommendations and directions for future research on the GJs subject.

## 2. Materials and Methods

**Figure 1:**
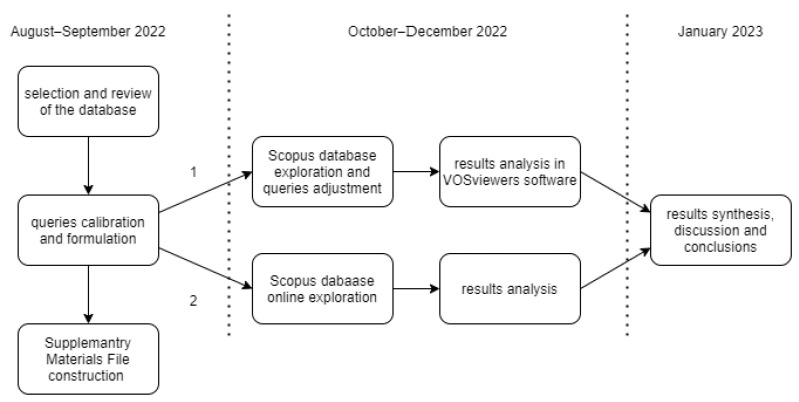
Methods and stages of the research. Source: Authors’ elaboration.

There are two complementary methods used in this research. First is the SLR variation with queries (Figure 1, path no. 1), and it is used to explore the scientific database to determine GJ’s definition consistency by the queries syntax. The second method is the search results analysis performed in the Scopus database online to identify the most cited publications and most contributing authors (Figure 1, path no. 2). The first method results are analyzed in VOSviewer in form of bibliometric maps. The second method is the analysis of the results in Scopus and is a development of the first used in this paper method, the SLR results method. Therefore, this section is divided into two subchapters.

The stages of the research covered 5 months of the bibliometric study. In the first period, based on the initial query formulation and calibration, observations have been made. In this stage, Supplementary Materials File S1 has been constructed. The conclusions and remarks have been gathered after all stages were completed and have been introduced into proper sections of this paper. 

### 2.1. Structured Literature Review Method Variation

The SLR method variation is supported by the research queries exploring the Scopus database. The SLR has its own procedure, which is not presented in detail in this paper [53,54]. In this study, the SLR method is used as a tool for the identification of knowledge gaps and the future direction of the research collected in bibliographic databases [55]. The SLR method variation is based on the research queries, which are used to explore the Scopus database. In this paper, the whole Scopus database was researched without any limitation to specific years or periods. The subject of this study is metadata of scientific literature collected in the Scopus database. This database was selected due to its broad scientific recognition and wider collection of content than other databases [55,56]. The information related to the bibliometric records of the Scopus database were explored by the bibliometric visualization tool software. The results of this method are presented in form of bibliometric maps with the use of the VOSviewer program (version 1.6.18). This software is commonly used by researchers in bibliometric studies in different research areas [57,58]. The method used in this research is to perform bibliometric analysis to produce a network visualization of keywords for the queries. In the variation of the SLR method, the three original queries were formulated and developed as presented in Table 1. There are differences in the formulated queries, although the queries have a syntax that corresponds with the database on which they are used. There are different numbers of results depending on the number of used green job equivalents and variants such as green employment, green collar, environmental job, or sustainable job.

The data presented in Table 1 and Table 2 queries’ construction and calibration are presented in Supplementary Materials File S1 in detail. These queries are focused on the GJs typologies visible in Scopus database-indexed publications. The subject areas in this research were not chosen automatically by the selection in the Scopus database but each paper was carefully reviewed to exclude misleading results to be further analyzed. This time-consuming operation explains the different dates presented in Table 1 and Table 2 results. There are differences in the formulated queries in Table 2, although the queries have a syntax that corresponds with the database on which they are used [59].

Presented queries do not differ in the publication type, years, or category, because such filters were not used to explore the Scopus database. The results obtained from Queries 4 and 5 (Q4 and Q5, respectively) were downloaded each time as a set of files in .csv format and during the export procedure, all fields on the publication were marked. Further analyses were carried out on the collected data in the VOSviewer program and the results are shown in bibliometric maps [60]. 

This research has its own limitations because the choice of the number of co-occurrences determines the result obtained in its graphical presentation and bibliometric map clarity. Therefore, a minimum number of 10 keyword co-occurrences was set for each bibliometric map initially, then it was changed and indicated specifically before each figure with the bibliometric map. The VOSviewer program allows researchers to define the research gaps covered by the published paper and indicate the directions of scientific development [55]. The exploration of the scientific database, presented in Table 2 Q4 and Q5, leads to the comparison of the GJ variants in two sets of query results.

### 2.2. Search Results Analysis in Scopus

Search results analysis in Scopus is a continuation of the SLR method. This analysis was based on Query 1 (symbol Q1 in Table 1), 1094 results, and was performed on the Scopus database online website after the option “analyze search results” was selected. The years 1966–2022 were the time frame for this online analysis. There were no other fields selected or deselected on the Scopus website.

## 3. Results

This section is divided into two subsections and reflects the two methods used in this research. The first subsection presents the bibliometric analysis which is gaining in popularity and is based on the SLR modification with queries and VOSviewer software. The second subsection contains the simple search results analysis offered by the Scopus database among the results of the indexed documents.

### 3.1. Bibliometric Analysis of SLR Method Results

Queries 4 and 5 (Table 2) were used for studying the Scopus database with different results for the same time point. The obtained results of those two queries were 671 and 611 publications, respectively (Table 2). Those results were analyzed in the VOSviewer software in form of bibliometric maps representing the keywords frequently occurring together. 

**Figure 2:**
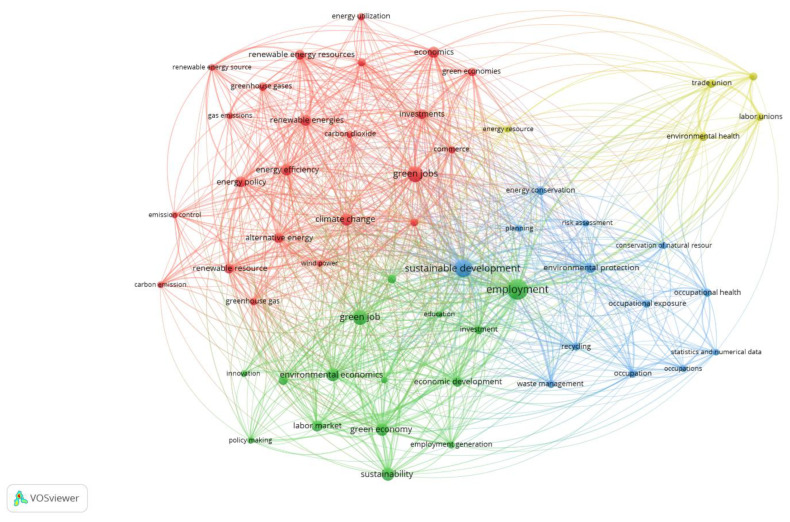
Bibliometric map of keywords co-occurrences Q4 results analysis in Scopus. Source: Authors’ elaboration.

Figure 2 is a bibliometric map of keyword co-occurrences of indexed keywords from publications index in Scopus distinguished as the Q4 results (Table 2). The method used to generate Figure 2 was full counting, and in this method 2418 indexed keywords were identified, among them 67 indexed keywords met the threshold of 10 co-occurrences. Among those results, keywords were referring to the countries and organizations’ names that were deselected from the proposed keywords list. Additionally deselected keywords from the proposed in VOSviewer list were: “human”, “humans”, “article”, “female”, “male”, and “adult”. Then from 67 keywords, 11 were deselected. Finally, there are 56 keywords collected in four clusters automatically colored and identified by the VOSviewer software. Figure 2 presents the keywords most often used in the scientific publications dedicated to green jobs, green collars, green employment, and sustainable employment and their combinations explored by the Q4 syntax. As a result, only two keywords representing “green jobs” and “green job” were placed in the bibliometric map (Figure 2). 

**Figure 3:**
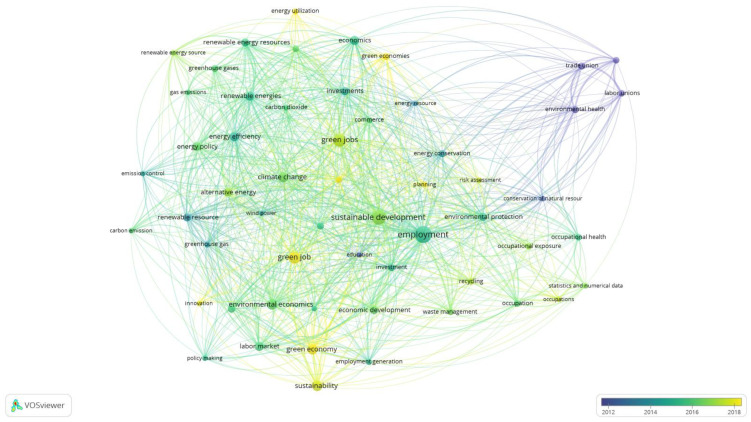
Overlay Visualization of keywords co-occurrences Q4 results analysis in Scopus. Source: Authors’ elaboration.

There are four clusters presented in Figure 2 and Table 3, and those clusters were automatically organized by the VOSviewer software. There are different numbers of keywords in each of the four clusters. In the first red-colored cluster there are 23 items and this is the most numerous group of keywords. The second is marked in green in Figure 3; this cluster consists of 15 keywords. The third cluster consists of 13 keywords presented in blue in Figure 2. There is also a yellow cluster with 5 automatically distinguished keywords. At this aggregate level, it is possible to identify themes of clusters of keywords based on the co-occurrence’s frequency. The size of nodes presented in Figure 2 is proportional to the number of occurrences of indexed keywords. Another important feature of the presented bibliometric map is the fact that closer proximity between nodes indicates a closer relationship between keywords. These characteristics allow aggregate keywords into clusters presented in Table 3. The number of occurrences for each keyword is indicated in parentheses, after each keyword.

Based on the generated results of Q4 there was also an overlay map generated (Figure 3). The purpose of this figure is to present the evolution of the scientific interests represented by the keywords related to the GJs. Figure 3 has automatically generated a time scale by VOSviewer. Figure 3 is similar to Figure 2 in shape and represents the same nodes, and edges as in Figure 2 and occurrences in Table 3.

In Figure 3 there are visible darker and lighter elements. The dark blue color represents the oldest keywords and this group reflexes the fourth cluster in Table 3. Keywords represented by the yellow nodes in Figure 3 represent the newest and still actual fields of interest in the subject of green jobs, and even the keyword “green job” is still in yellow in Figure 3. The importance of these yellow-marked keywords is the basis of the discussion and conclusions for future research directions in respective sections of this paper. 

**Figure 4:**
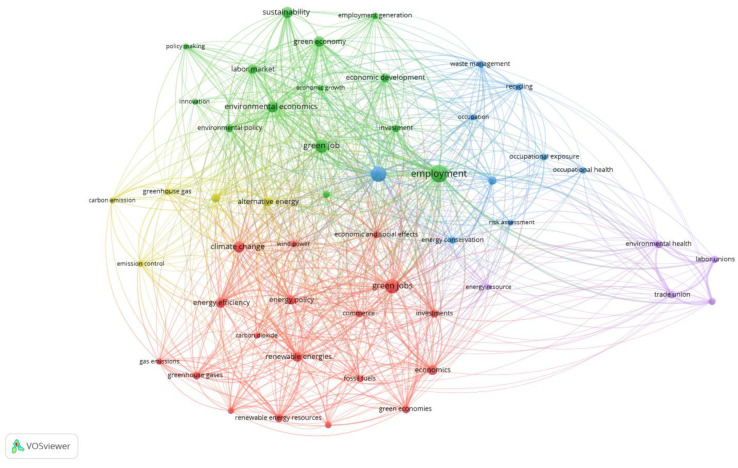
Bibliometric map of keywords co-occurrences Q5 results analysis in Scopus. Source: Authors’ elaboration.

Figure 4 is a bibliometric map of keywords co-occurrences of indexed keywords from publications index in Scopus distinguished as the Q5 results (Table 2). The method used to generate Figure 4 was full counting of indexed keywords co-occurrences, and in this method, 2213 indexed keywords were identified, among them 58 indexed keywords met the threshold of 10 co-occurrences. Among those results, keywords were referring to the countries and organizations’ names that were deselected from the proposed keywords list. Additionally deselected keywords from the proposed in VOSviewer list were: “human”, “humans”, and “article”. Then from 58 keywords, 7 were deselected. Finally, there are 51 keywords collected in four clusters, automatically colored and identified by the VOSviewer software. Figure 4 presents the keywords most often used in the scientific publications dedicated to green jobs, green collars, green employment, and sustainable employment and their combinations explored by the Q5 syntax. As result, only two keywords representing “green jobs” and “green job” were placed in the bibliometric map (Figure 4). 

There are five clusters presented in Figure 4 and described in Table 4 which are automatically colored by VOSviewer software. There is the most numerous of all clusters colored in red the first cluster with 18 items. Second is the green cluster with 14 distinguished keywords. The third is a blue cluster consists of 9 items and collects the keywords: “recycling”, “waste management”, “risk assessment”, “occupational exposure”, “occupational health”, occupation”, “environmental health”, “energy conservation”, and “sustainable development”. There is also a fourth yellow cluster with 5 items. The fifth cluster in Table 4, consists also of 5 keywords but it is colored purple. The number of occurrences for each keyword is indicated in parentheses, after each keyword, in Table 4.

There are similarities between the presented two tables with VOSviewer results automatically divided into clusters, although Table 3 consists of more keywords than Table 4. The number of clusters in Table 4 is also smaller than in Table 3. The first cluster in Table 4 revolves around negative aspects of the GJs definition expressed in human activities’ pressure on the natural environment measures. The second cluster presented in Table 4 consists of positive aspects of the GJs definition expressed in progress, economic development, and sustainability. There is also a third cluster in Table 4 and this cluster revolves around employee health protection and conservation of the resources. The fourth cluster presented in Table 4 represents the rules or regulations associated with the GJs which influence “energy resources”, “environmental health”, “labor unions”, “organization and management”, and “trade union”.

**Figure 5:**
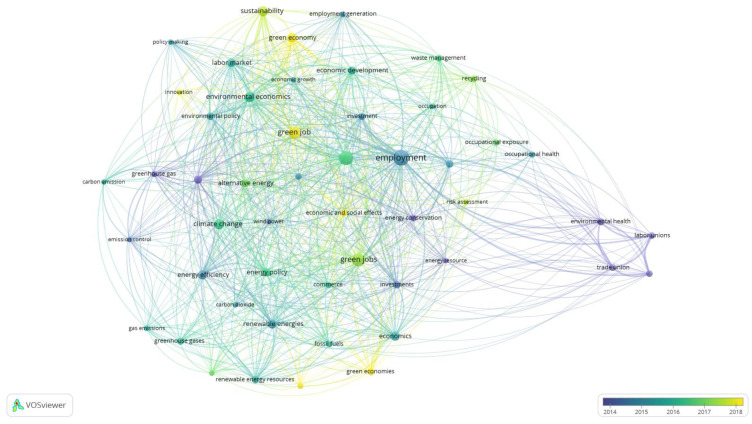
Overlay Visualization of keywords co-occurrences Q5 results analysis in Scopus. Source: Authors’ elaboration.

There are the same similarities between Figure 4 and Figure 5 as the described similarities between Figure 2 and Figure 3, in terms of the shape and connections. In Figure 5 there is also an automatically distinguished time scale of co-occurring keywords evolution. The oldest keywords marked in darker colors correspond with the fifth subnetwork of the created map and parts of the other clusters. The distribution of those older keywords is then complex. However, the lighter keywords representing the relatively newest scientific interests are scattered. In Figure 5, attention is deserved for two centrally located keywords “green job” and “green jobs” marked in lighter colors, which indicates the ongoing debate which revolves around those terms. Based on Figure 5, the view on the perspective and emerging future directions of studies are developed in the discussion and conclusion sections.

The order of keywords in each cluster is automatically proposed by the VOSviewer software. Their complex relations prove that the concept of GJs emerged from the concept of sustainable development and the assumption that greening the economy or creating a green economy, would generate GJs [61]. Therefore, the most important and biggest node in Figure 2 and Figure 3 is the “employment” keyword. Its central place in the bibliometric map reflects the research results, which claim that with GJs it is possible to fight unemployment as well as to counteract environmental degradation [62].

There are not only quantitative differences in Q4 and Q5 results but also qualitative, related to the GJs definition. The broad definition of GJs has raised concerns among the expert teams and created the need to clarify the direction of further work on the definition of GJs. As part of their work on the GJs definition, the teams identified their specific sectors for their regions that meet the condition of respecting nature’s assets and residents [63,64]. The colors presented in Figure 2 and Figure 4 are also different, although the keywords in Table 3 and Table 4 are similar. The shapes of Figure 2 and Figure 4 are also matching. The most interesting feature of both figures is separated on the right side of the area of the figures which consists of five keywords. These nodes are as follows: “energy resource”, “environmental health”, “labor unions”, “organization and management”, and “trade union”. These keywords are mainly related to the green labor market or labor conditions and are the same in both bibliometric maps.

### 3.2. Search Results Analysis in Scopus

The analysis performed online on the Scopus website was based on 1094 documents indexed in Scopus scientific database, which are Q1 results (Table 1). The first publication among these results and related to the GJs was an article by Ronald J. Burke, titled *Are Herzberg’s motivators and hygienes unidimensional?* published in the 1966 Journal of Applied Psychology [65]. This publication used the term “environmental job” and research revolved around job satisfaction or dissatisfaction in a such-named green job [65]. Therefore, the time frame of the Scopus exploration starts in 1966.

**Figure 6:**
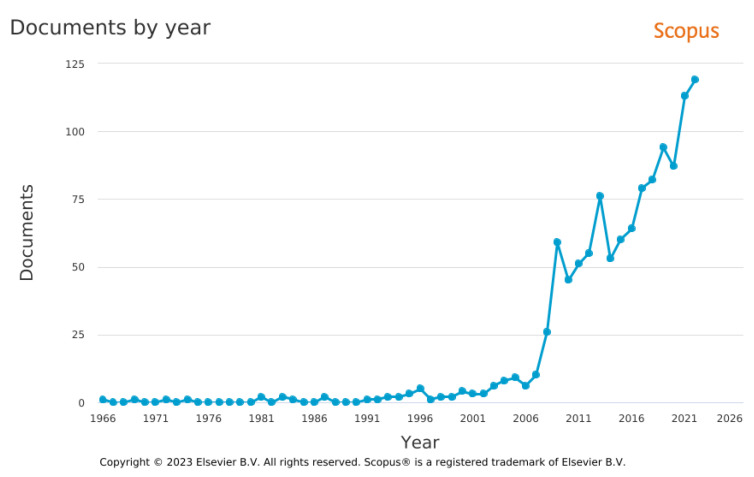
Documents results of Q1 in Scopus. Source: Authors’ elaboration.

Figure 6 presents the rapid growth in the amount of indexed publications dedicated to GJs or their variant names (as indicated in Q1 syntax) that occurred in 2008 (with 26 publications). Since then, the linear trend is growing, and 2022 was the year in which the highest number of publications (119 documents in Scopus) dedicated to GJs were published.

**Figure 7:**
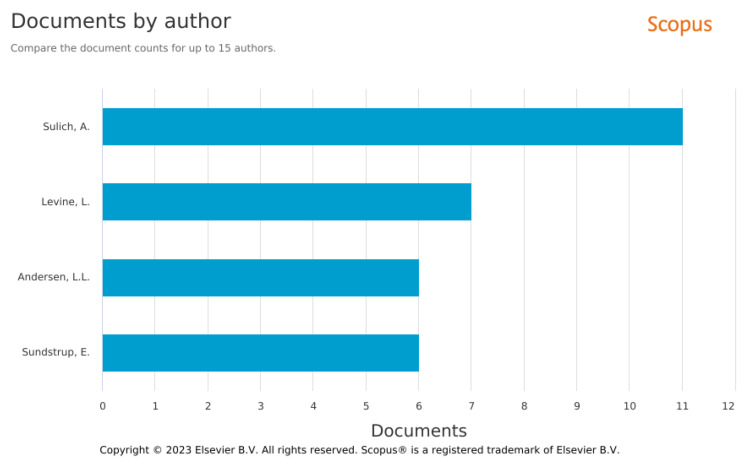
Documents by authors. Analysis of the results of Q1 in Scopus. Source: Authors elaboration.

The analysis of the Q1 result allowed for identification of the main quantitatively contributing authors in the field of GJs by a number of authored documents collected in Scopus. In Figure 7 are presented selected authors with six and more publications indexed in Scopus. There is a similar analysis result for the other queries [66].

The authors who contributed most to the subject of GJs (Figure 7) are not the most-cited authors. Those are presented in Table 5, where the most cited publications (over 400 citations in Scopus) among results of Q1 are also listed.

The Publications presented in Table 5 revolve around different name variants for GJs. The first publication titled *Comparing structural and index decomposition analysis* uses the term environmental employment [67]. The second publication is related to sustainable jobs and explores smart manufacturing subjects [68]. The only book among the most cited publications is dedicated to the GJs concept and labor market issues. In his book, Ross surveys “the new topography of the global workplace and finds an emerging pattern of labor instability and uneven development on a massive scale” [69]. The fourth of the most cited publications indexed in Scopus does not refer to any of explored GJs variants proposed in queries employed in this article. The article published in the Energy Policy journal presents an analytical job creation model for the USA power sector from 2009 to 2030 [46]. Therefore, all works gathered in Table 5 combine non-fossil fuels technologies, resource-efficient economy, and technologies of carbon capture and storage with positive changes in the labor market expressed by GJs creation [37,70]. The different qualitative approaches lead to new implications in the number of created jobs related to green and sustainable economic transformation.

## 4. Discussion

Based on the analyses carried out, it is important to note the lack of uniform naming of GJs, which is overlooked by some of the researchers of this problem. As a result, the resulting analyses are not fully comprehensive; however, they present the most popular keywords associated with the GJs. The existing used terms of GJs, which are presented in the third part, indicate the evolution of this concept and the slowly establishing pattern in the literature of the name GJs, which is the most frequently cited name of the explored phenomenon. Nevertheless, there are still many researchers who use quotation marks to refer to this type of job or a completely different name in the form of various equivalents of the names: green employment [71,72], green collar [73,74], environmental job [75], or sustainability job [76]. The results show that the use of terms equivalent to green jobs depends on the context of their use. Green collars appear in engineering articles on renewable energy technologies. Sustainability jobs, on the other hand, appear in articles related to strategy formulation. Authors of publications seeking scientific novelty create new names for already defined terms, which is why constructs such as “ecojobs” appear [77]. 

Emerging new concepts make it difficult to carry out a comprehensive analysis of the concept of green jobs and expand the context for searching databases. Therefore, the analysis undertaken focuses on commonly occurring synonyms for green jobs. The analysis undertaken is devoid of the mantle of linguistic research or somatic analysis of words as undertaken by the authors of the publications under investigation. At the same time, it may be an interesting new direction for future research, which may show the different perceptions of researchers regarding green jobs. Such different nomenclature forces researchers on the subject of GJs to analyze the content of individual articles quite meticulously at the stage of selection for analysis, which is carried out using programs such as VOSviewer. Researchers should especially pay attention to “green collar”, a name that also appears concerning issues related to military areas (with the absence here of any reference to sustainability issues). This forces researchers to perform an in-depth qualitative analysis of the surveyed publications when qualifying them for analysis using VOSviewer. Only after such a qualitative analysis that leads to the exclusion of articles not related to the topic of sustainability the procedure presented in Figure 1 can be applied.

The year 2008 was linked to the global economic crisis [78]. The study observed a multidimensionality of social economic and environmental problems, which were closely interlinked. In this context, solutions were sought to find a way out of the crisis. It was noted that the current paradigm of economic development, which is mainly based on non-renewable resources, is unreliable and does not provide opportunities for future generations [77,79]. The researchers, therefore, drew attention to the need to implement sustainable development and related green jobs into economic practice [80,81]. This explains the growing interest in green jobs, not only from a scientific but also from a practical point of view [82].

A limitation of this study is the lack of a detailed dynamic analysis performed in VOSviewer which addresses the strength of the connections between individual keywords. Such a dynamic analysis is only possible when using the VOSviewer program and its graphical representation is impossible, due to a large number of identified connections (graph edges). However, the lack of such an analysis does not affect the quality of the presented research results and conclusions.

It should be recognized that GJs are a key element of the green economy. They bind numerous areas of the economy together with their issues. Based on the analysis carried out, it can be concluded that when discussing the issue of GJs, an important emphasis is placed on the “education” keyword, which is located close to “employment” in a green cluster in Figure 2. To the “education” keyword there are other related terms such as knowledge, skills, attitudes [83,84], and pro-environmental behavior of employees researched deeply in Green Humans Resources [29,85]. These are the elements of complex business activities that indicate whether or not an employee employed at a given green job will contribute to minimizing the negative impact of a given economic entity on the environment [84]. This area was indicated also automatically by the bibliometric software as the first and most numerous cluster in both analyses of Queries 4 and 5.

## 5. Conclusions

In this paper 671 (Q4 in Table 2) and 611 (Q5 in Table 2) peer-reviewed academic publications associated with green jobs (GJs) using Scopus were identified. This study used the VOSviewer software to present a bibliometric analysis and to identify key areas around which the topic of GJs revolves in the literature. Then, identified clusters of keywords were associated with different aspects of green jobs. The very separation of GJs from the total number of jobs contributes to the segmentation of the labor market. The authors would like to emphasize that GJs form a key element of the green labor market [86]. At the same time, based on the analyzed scientific studies, it can be seen that due to the fact of gradual greening of the economy, not all employees who would like to work in GJs will find such employment, despite having the appropriate green competencies and qualifications, as well as being characterized by pro-environmental behavior and attitudes. Hence, to ensure that their green capital is not depreciated based on non-GJs, an alternative is to take up green self-employment. This area should be recognized by policymakers influencing the creation of national policies on labor market issues [87]. The active labor market policy instruments should be developed in the context of supporting green self-employment [63].

The creation of GJs contributes to the need to implement an appropriate management process in business entities so that green human capital is properly used to achieve competitive advantages [88]. In addition, proper management of green human capital in business entities is aimed at preventing its depreciation. Hence, one should notice the emerging theme of green human resource management, or sustainable human resource management, in the scientific studies analyzed. Thus, the emergence of GJs not only causes effects of an economic nature (the need to commit adequate capital to green existing jobs or create new GJs), but also strictly organizational in terms of the need to develop a new model of human capital management in the organization.

## Figures and Tables

**Table 1:** Queries focused on typologies used in the Scopus scientific database exploration on 20 August 2022.

Symbol	Query Syntax	No. of Results (20 August 2022)
Q1	TITLE-ABS-KEY (“green job” OR “green employment” OR “green collar” OR “sustainable job” OR “sustainable employment” OR “sustainability job” OR “eco-friendly employment” OR “eco employment” OR “environmental job” OR “environmental employment”)	1094
Q2	TITLE-ABS-KEY (“green job” OR “green employment” OR “green collar” OR “sustainability job” OR “eco-friendly employment” OR “eco employment” OR “environmental job” OR “environmental employment”)	702
Q3	TITLE-ABS-KEY (“green job” OR “green employment” OR “green collar” OR “sustainability job” OR “environmental job”)	684

Source: Authors’ elaboration.

**Table 2:** Syntaxes used in Queries after calibration for the Scopus scientific database exploration GJs concept.

No.	Query Syntax	No. of Results (31 December 2022)
Q4	TITLE-ABS-KEY ({green job} OR {green jobs} OR {green-jobs} OR {‘green’ job} OR {‘green’ jobs} OR {‘green job’} OR {‘green jobs’} OR {green collar} OR {green-collar} OR {‘green-collar’} OR {‘green collar’} OR {green employment} OR {green employments} OR {environmental job} OR {environmental jobs} OR {‘environmental job’} OR {sustainability job})	671
Q5	TITLE-ABS-KEY ({green job} OR {green jobs} OR {*green job*} OR {green-jobs} OR {‘green’ job} OR {‘green’ jobs} OR {‘green job’} OR {‘green jobs’} ) OR ({green collar} {green collars} OR {*green collar*} OR {green-collar} OR {‘green-collar’} OR {‘green collar’} OR {green employment} OR {*green employment*} OR {green employments}) OR ({environmental job} OR {environmental jobs} OR {*environmental job*} OR {‘environmental job’} OR {environm* employment}) OR ({sustainab* job} OR {sustainab* employment} OR {environm* employment})	611

Source: Authors’ elaboration.

**Table 3:** Clusters of keyword co-occurrences presented in Figure 2 for Scopus Q4.

Cluster	Color	Keywords
1	Red	alternative energy (23), carbon dioxide (12), carbon emission (10), climate change (34), commerce (13), economic and social effects (14), economics (28), emission control (10), energy efficiency (28), energy policy (24), energy utilization (12), fossil fuels (14), gas emissions (11), green economies (18), green jobs (59), greenhouse gas (13), greenhouse gases (17), investments (21), renewable energies (28), renewable energy resources (21), renewable energy source (10), renewable resource (22), wind power (11)
2	Green	economic development (23), economic growth (11), education (10), employment (96), employment generation (13), environmental economics (36), environmental impact (17), environmental policy (20), green economy (33), green job (48), innovation (11) investment (18), labor market (27), policy making (10), sustainability (36)
3	Blue	conservation of natural resources (10), energy conservation (16), environmental protection (21), occupation (15), occupational exposure (14), occupational health (15), occupations (11), planning (11), recycling (15), risk assessment (11), statistics and numerical data (10), sustainable development (72), waste management (14)
4	Yellow	energy resource (10), environmental health (17), labor unions (15), organization and management (14), trade union (16)

Source: Authors’ elaboration.

**Table 4:** Clusters of keywords co-occurrences visible in Figure 4 for Scopus Q5 results.

Cluster	Color	Keywords
1	Red	carbon dioxide (11), climate change (33), commerce (13), economic and social effects (14), economics (27), energy efficiency (27), energy policy (22), energy utilization (12), fossil fuels (13), gas emissions (18), green economies (18), green jobs (59), greenhouse gases (17), investments (19), renewable energies (28), renewable energy resource (20), renewable energy sources (21), wind power (11)
2	Green	economic development (22), economic growth (11), employment (88), employment generation (13), environmental economics (35), environmental impact (14), environmental policy (19), green economy (31), green job (48), innovation (11), investment (18), labor market (26), policy making (10), sustainability (33)
3	Blue	energy conservation (15), environmental protection (19), occupation (11), occupational exposure (13), occupational health (12), recycling (14), risk assessment (11), sustainable development (67), waste management (14)
4	Yellow	alternative energy (23), carbon emission (10), emission control (10), greenhouse gas (13), renewable resource (22)
5	Purple	energy resource (10), environmental health (17), labor unions (15), organization and management (14), trade union (16)

Source: Authors’ elaboration.

**Table 5:** Top Four most cited publications dedicated to the subject of GJs.

Document Type	Document Title	Authors	Source	Cited by
Journal article	Comparing structural and index decomposition analysis [67]	Hoekstra, R., van der Bergh, J.J.C.J.M.	Energy Economics, 2003, 25(1), pp. 39–64	620
Journal article	Smart manufacturing, manufacturing intelligence and demand-dynamic performance [68]	Davis, J., Edgar, T., Porter, J., Bernaden, J., Sarli, M.	Computers and Chemical Engineering, 2012, 47, pp. 145–156	481
Book	Nice work if you can get it: Life and labor in precarious times [69]	Ross, A.	Nice work if you can get it: Life and labor in precarious times, New York University Press, New York, 2009, pp. 1–263	432
Journal article	Putting renewables and energy efficiency to work: How many jobs can the clean energy industry generate in the US? [46]	Wei, M., Patadia, S., Kammen, D.M.	Energy Policy, 2010, 38(2), pp. 919–931	418

Source: Authors’ elaboration.

## Data Availability

Not applicable.

## Supplementary Materials

The following supporting information can be downloaded at https://www.mdpi.com/article/10.3390/ijerph20042886/s1. Table S1. Syntaxes used in Queries calibration for the Scopus scientific database exploration variants of naming green job; Table S2. Syntaxes used in Queries calibration for the Scopus scientific database exploration variants of naming green collar; Table S3. Syntaxes used in Queries calibration for the Scopus scientific database exploration variants of naming green employment; Table S4. Syntaxes used in Queries calibration for the Scopus scientific database exploration variants of naming environmental job; Table S5. Syntaxes used in Queries calibration for the Scopus scientific database exploration variants of naming sustainability job.

## References

[1] Wang C. (2015). The assessment of the green transformation capacity in low carbon economy of China. Open Cybern. Syst. J..

[2] Cheba K., Bąk I., Szopik-Depczyńska K., Ioppolo G. (2022). Directions of green transformation of the European Union countries. Ecol. Indic..

[3] Sulich A., Sołoducho-Pelc L. (2022). The circular economy and the Green Jobs creation. Environ. Sci. Pollut. Res..

[4] Lehr U., Lutz C., Edler D. (2012). Green jobs? Economic impacts of renewable energy in Germany. Energy Policy.

[5] Mohan Das Gandhi N., Selladurai V., Santhi P. (2006). Unsustainable development to sustainable development: A conceptual model. Manag. Environ. Qual. An Int. J..

[6] Agbedahin A.V. (2019). Sustainable development, Education for Sustainable Development, and the 2030 Agenda for Sustainable Development: Emergence, efficacy, eminence, and future. Sustain. Dev..

[7] Rutkowska-Podołowska M., Sulich A., Howaniec H., Malara Z., Wyród-Wróbel J. (2016). Zielone miejsca pracy w gospodarce [Green Jobs in Economy]. Improving the Efficiency in Eterprise—Selected Aspects.

[8] Mróz-Gorgoń B., Wodo W., Andrych A., Caban-Piaskowska K., Kozyra C. (2022). Biometrics Innovation and Payment Sector Perception. Sustainability.

[9] Kozar Ł.J. (2019). Zielone Miejsca Pracy. Uwarunkowania–Identyfikacja–Oddziaływanie na Lokalny Rynek Pracy; [Green Jobs. Determinants-Identification-Impact on the Local Labour Market].

[10] Hou J., Teo T.S.H., Zhou F., Lim M.K., Chen H. (2018). Does industrial green transformation successfully facilitate a decrease in carbon intensity in China? An environmental regulation perspective. J. Clean. Prod..

[11] Sulich A., Sołoducho-Pelc L. (2022). Changes in Energy Sector Strategies: A Literature Review. Energies.

[12] Sulich A., Zema T. (2018). Green jobs, a new measure of public management and sustainable development. Eur. J. Environ. Sci..

[13] DeVries H., Tripoli L. (2010). Corporate progress: What are green jobs paying?. Sustainability.

[14] Pavlović M., Vulić M., Pavlović A. (2019). Circular Economy in Rrepublic of Serbia and Region.

[15] Dordmond G., de Oliveira H.C., Silva I.R., Swart J. (2021). The complexity of green job creation: An analysis of green job development in Brazil. Environ. Dev. Sustain..

[16] Sulich A., Rutkowska M., Popławski Ł. (2020). Green jobs, definitional issues, and the employment of young people: An analysis of three European Union countries. J. Environ. Manag..

[17] Luca F.-A., Epuran G., Ciobanu C.-I., Horodnic A.V. (2019). Green jobs creation—Main element in the implementation of bioeconomic mechanisms | Crearea de locuri de muncă ecologice—Componentă de bază pentru implementarea mecanismelor bioeconomice. Amfiteatru Econ..

[18] Pop O., Dina G.C., Martin C. (2011). Promoting the corporate social responsibility for a Green Economy and innovative jobs. Procedia Soc. Behav. Sci..

[19] Aceleanu M.I. (2015). Green jobs in a green economy: Support for a sustainable development. Prog. Ind. Ecol..

[20] van der Ree K. (2019). Promoting Green Jobs: Decent Work in the Transition to Low-Carbon, Green Economies. Rev. Int. Polit. Développement.

[21] Falxa-Raymond N., Svendsen E., Campbell L.K. (2013). From job training to green jobs: A case study of a young adult employment program centered on environmental restoration in New York City, USA. Urban For. Urban Green..

[22] Wagner C. (2013). Adult Learning Meets the Green Economy: Lessons From a Green Jobs Education Project. Adult Learn..

[23] Heong Y.M., Sern L.C., Kiong T.T., Binti Mohamad M.M. (2016). The Role of Higher Order Thinking Skills in Green Skill Development. MATEC Web Conf..

[24] Cabral C., Dhar R.L. (2021). Green competencies: Insights and recommendations from a systematic literature review. Benchmarking.

[25] Ibrahim Z., Lai C.S., Zaime A.F., Lee M.F., Othman N.M. (2020). Green skills in knowledge and attitude dimensions from the industrial perspective. IOP Conf. Ser. Mater. Sci. Eng..

[26] Renwick D.W.S., Jabbour C.J.C., Muller-Camen M., Redman T., Wilkinson A. (2016). Contemporary developments in Green (environmental) HRM scholarship. Int. J. Hum. Resour. Manag..

[27] Piwowar-Sulej K., Mroziewski R., Bachnik K., Kaźmierczak M., Rojek-Nowosielska M., Stefańska M., Szumniak-Samolej J. (2022). Management by Values and Socially Responsible HRM as Success Factors in the Time of the COVID-19 Crisis. Corporate Social Responsibility and Sustainability.

[28] Murga-Menoyo M.Á. (2014). Learning for a sustainable economy: Teaching of green competencies in the university. Sustainability..

[29] Kozar Ł. (2017). Shaping the Green Competence of Employees in an Economy Aimed at Sustainable Development. Green Hum. Resour. Manag..

[30] Consoli D., Marin G., Marzucchi A., Vona F. (2016). Do green jobs differ from non-green jobs in terms of skills and human capital?. Res. Policy.

[31] Bowen A., Kuralbayeva K., Tipoe E.L. (2018). Characterising green employment: The impacts of ‘greening’ on workforce composition. Energy Econ..

[32] Stukalo N., Simakhova A. (2019). Social Dimensions of Green Economy. Filos. Sociol..

[33] Kozar Ł.J., Matusiak R., Paduszyńska M., Sulich A. (2022). Green Jobs in the EU Renewable Energy Sector: Quantile Regression Approach. Energies.

[34] Sulich A., Soliman K.S. (2018). The Green Economy Development Factors. Vision 2020: Sustainable Economic Development and Application of Innovation Management from Regional Expansion to Global Growth. Proceedings of the 32nd International Business Information Management Association Conference, Sevilla, Hiszpania, 15–16 November 2018.

[35] Burger M., Stavropoulos S., Ramkumar S., Dufourmont J., van Oort F. (2019). The heterogeneous skill-base of circular economy employment. Res. Policy.

[36] Dell’Anna F. (2021). Green jobs and energy efficiency as strategies for economic growth and the reduction of environmental impacts. Energy Policy.

[37] Kozar Ł. (2016). “Green” jobs by sector of the economy; [“Zielone” miejsca pracy w ujęciu sektorowym gospodarki]. Ekonomia Zrównoważonego Rozwoju. Społeczeństwo, Środowisko, Innowacje w Gospodarce.

[38] Unay-Gailhard İ., Bojnec Š. (2019). The impact of green economy measures on rural employment: Green jobs in farms. J. Clean. Prod..

[39] Annandale D., Morrison-saunders A., Duxbury L. (2004). Regional sustainability initiatives: The growth of green jobs in Australia. Local Environ..

[40] Stoyanova Z., Harizanova H. (2017). Analysis of the External Environment of Green Jobs in Bulgaria. Econ. Altern..

[41] Ones D.S., Dilchert S., Huffman A.H., Klein S.R. (2013). Measuring, Understanding, and Influencing Employee Green Behaviors. Green Organizations: Driving Change with I-O Psychology.

[42] Kozar Ł. (2022). Która ze stosowanych metod identyfikacji zielonych miejsc pracy w gospodarce jest najefektywniejsza? [Which of the methods used to identify green jobs in the economy is the most effective?]. W Poszukiwaniu Zielonego Ładu.

[43] Bowen A., Kuralbayeva K. (2015). Looking for Green Jobs: The Impact of Green Growth on Employment. Grantham Research Institute on Climate Change and the Environment and Global Green Growth Institute Working Papers. http://www.lse.ac.uk/grantham/.

[44] Böhringer C., Rivers N.J., Rutherford T.F., Wigle R. (2012). Green jobs and renewable electricity policies: Employment impacts of Ontario’s feed-in tariff. B.E. J. Econ. Anal. Policy.

[45] Tănasie A.V., Năstase L.L., Vochița L.L., Manda A.M., Boțoteanu G.I., Sitnikov C.S. (2022). Green Economy—Green Jobs in the Context of Sustainable Development. Sustainability.

[46] Wei M., Patadia S., Kammen D.M. (2010). Putting renewables and energy efficiency to work: How many jobs can the clean energy industry generate in the US?. Energy Policy.

[47] Kammen D.M., Engel D. Green Jobs and the Clean Energy Economy. Proceedings of the 2009 UN Climate Change Conference (COP15).

[48] Ge Y., Zhi Q. (2016). Literature Review: The Green Economy, Clean Energy Policy and Employment. Energy Procedia.

[49] Stevis D., Räthzel N., Jackson T., Uzzell D. (2012). Green jobs? Good Jobs? Just Jobs? US Labour Unions Confront Climate Change1. Trade Unions in the Green Economy. Working for the Environment.

[50] Bogusz K., Sulich A., Soliman K.S. (2019). The Sustainable Development Strategies in Mining Industry. Education Excellence and Innovation Management through Vision 2020.

[51] García Vaquero M., Sánchez-Bayón A., Lominchar J. (2021). European Green Deal and Recovery Plan: Green Jobs, Skills and Wellbeing Economics in Spain. Energies.

[52] Stanef-Puică M.-R., Badea L., Șerban-Oprescu G.-L., Șerban-Oprescu A.-T., Frâncu L.-G., Crețu A. (2022). Green Jobs—A Literature Review. Int. J. Environ. Res. Public Health.

[53] Zema T., Sulich A., Grzesiak S. (2022). Charging Stations and Electromobility Development: A Cross-Country Comparative Analysis. Energies.

[54] Boyack K., Glänzel W., Gläser J., Havemann F., Scharnhorst A., Thijs B., van Eck N.J., Velden T., Waltmann L. (2017). Topic identification challenge. Scientometrics.

[55] Zema T., Sulich A. (2022). Models of Electricity Price Forecasting: Bibliometric Research. Energies.

[56] Weron R. (2014). Electricity price forecasting: A review of the state-of-the-art with a look into the future. Int. J. Forecast..

[57] Bertocci F., Mannino G. (2022). Can Agri-Food Waste Be a Sustainable Alternative in Aquaculture? A Bibliometric and Meta-Analytic Study on Growth Performance, Innate Immune System, and Antioxidant Defenses. Foods.

[58] Gizzi F.T. (2015). Worldwide trends in research on the San Andreas Fault System. Arab. J. Geosci..

[59] Elsevier How Can I Best Use the Advanced Search?—Scopus: Access and Use Support Center. https://service.elsevier.com/app/answers/detail/a_id/11365/supporthub/scopus/#tips.

[60] van Eck N.J., Waltman L. (2010). Software survey: VOSviewer, a computer program for bibliometric mapping. Scientometrics.

[61] Baer P., Brown M.A., Kim G. (2015). The job generation impacts of expanding industrial cogeneration. Ecol. Econ..

[62] Manijean L., Saffache P. (2017). Geothermal energy: The pool of jobs!!. Trans. Geotherm. Resour. Counc..

[63] Ramos D., Afonso P., Rodrigues M.A. (2020). Integrated management systems as a key facilitator of occupational health and safety risk management: A case study in a medium sized waste management firm. J. Clean. Prod..

[64] Rutkowska M., Sulich A., Szczygieł N., Kovářová E., Melecký L., Staníčková M. (2016). Green jobs. Proceedings of the 3rd International Conference on European Integration 2016, ICEI 2016.

[65] Burke R.J. (1966). Are Herzberg’s motivators and hygienes unidimensional?. J. Appl. Psychol..

[66] Sulich A. Green Jobs Impact by Adam Sulich. https://www.researchgate.net/publication/366445592_Green_jobs_impact_by_Adam_Sulich?channel=doi&linkId=63a217ed9835ef259035a189&showFulltext=true.

[67] Hoekstra R., van der Bergh J.J.C.J.M. (2003). Comparing structural and index decomposition analysis. Energy Econ..

[68] Davis J., Edgar T., Porter J., Bernaden J., Sarli M. (2012). Smart manufacturing, manufacturing intelligence and demand-dynamic performance. Comput. Chem. Eng..

[69] Ross A. (2009). Nice Work If You Can Get It: Life and Labor in Precarious Times.

[70] Kulhanek L., Sulich A., Zema T., Stanickova M., Melecky L. (2022). European integration and real convergence in V4 Group: Transformation towards green economy. Proceedings of the 6th International Conference on European Integration.

[71] Elliott R.J.R., Lindley J.K. (2017). Environmental Jobs and Growth in the United States. Ecol. Econ..

[72] Hersh M. (2015). Ethical Engineering for International Development and Environmental Sustainability.

[73] Sullivan J., Lee C.-Y. (2018). A New Era in Democratic Taiwan: Trajectories and Turning Points in Politics and Cross-Strait Relations.

[74] Lee J.-H., Lee W., Yoon J.-H., Seok H., Roh J., Won J.-U. (2015). Relationship between symptoms of dry eye syndrome and occupational characteristics: The Korean National Health and Nutrition Examination Survey 2010–2012. BMC Ophthalmol..

[75] Ben Cheikh N., Ben Zaied Y. (2020). Nonlinear analysis of employment in waste management. Appl. Econ. Lett..

[76] Rempel G., Moshiri M., Milligan C., Bittner J., Montufar J. (2012). Options for Hauling Fully Loaded ISO Containers in the United States. J. Transp. Eng..

[77] Song K., Kim H., Cha J., Lee T. (2021). Matching and Mismatching of Green Jobs: A Big Data Analysis of Job Recruiting and Searching. Sustainability.

[78] Baciu E.-L. (2022). Employment Outcomes of Higher Education Graduates from during and after the 2007–2008 Financial Crisis: Evidence from a Romanian University. Sustainability.

[79] Bracarense N., Bracarense Costa P.A. (2022). Green Jobs: Sustainable Path for Environmental Conservation and Socio-Economic Stability and Inclusion. Rev. Polit. Econ..

[80] Sylvan A.M. (2014). Social Innovation, Entrepreneurship and New Green Jobs: Successful Experiences in Mexico.

[81] Saba A. (2017). Five year review: Evolving environmental law and advice to green lawyers. Environ. Claims J..

[82] Knuth S. (2019). Whatever Happened to Green Collar Jobs? Populism and Clean Energy Transition. Ann. Am. Assoc. Geogr..

[83] Junhong W. (2021). Education model of guiding college students to find employment in environmental company. J. Environ. Prot. Ecol..

[84] Kozar Ł., Oleksiak P. (2022). Organizacje Wobec Wyzwań Zrównoważonego Rozwoju—Wybrane Aspekty [Organisations Facing the Challenges of Sustainable Development—Selected Aspects].

[85] Sulich A., Rutkowska-Podołowska M., Borowiecki R., Kaczmarek J. (2017). Green jobs and changes in modern economy on the labour market. The Propensity To Changes in the Competitive and Innovative Economic Environment: Processes–Structures–Concepts.

[86] Rutkowska M., Sulich A. (2020). Green Jobs on the background of Industry 4.0. Procedia Comput. Sci..

[87] Budur T., Demir A. (2022). The Relationship Between Transformational Leadership and Employee Performance: Mediating Effects of Organizational Citizenship Behaviors. Iran. J. Manag. Stud..

[88] Sołoducho-Pelc L., Sulich A. (2020). Between Sustainable and Temporary Competitive Advantages in the Unstable Business Environment. Sustainability.

