# Supplementary material for: Green Jobs: Bibliometric Review
Source: Int J Environ Res Public Health. 2023 Feb 7;20(4):2886. doi: 10.3390/ijerph20042886 (PMC9961274; doi:10.3390/ijerph20042886)
Supplement: Supplementary file 1 [file ijerph-20-02886-s001.zip › ijerph-2193337-supplementary.pdf]

Supplementary Materials File S1

Łukasz Jarosław Kozar [1,*] and Adam Sulich [2,3,*]

1 Department of Labour and Social Policy, Faculty of Economics and Sociology, University of Lodz, ul. Rewolucji 1905 r. no 37, 90-214 Lodz, Poland
2 Department of Advanced Research in Management, Faculty of Business Management, Wroclaw University of Economics and Business, ul. Komandorska 118/120, 53-345 Wroclaw, Poland
3 Schulich School of Business, York University, 4700 Keele Street, Toronto, ON M3J 1P3, Canada
* Correspondence: lukasz.kozar@uni.lodz.pl (Ł.J.K.); adam.sulich@ue.wroc.pl (A.S.)

This file contains 5 Tables with original query results used for calibration and development of queries used in the paper titled Green Jobs: Bibliometric Review. This appendix contains results data for 10 September 2022 obtained in the Scopus database. The tables' results differ due to the different operators and keywords used in the Scopus database search. Presented results in Tables S1-S5 differ also between raw query results and those accepted after indexed works reading (marked with ♠ asterix in tables).

Table S1. Syntaxes used in Queries calibration for the Scopus scientific database exploration variants of naming green job

Query Symbol	Query Syntax	No. of results (10 September 2022)
Q1_GJ	TITLE-ABS-KEY ({green job})	122
Q2_GJ	TITLE-ABS-KEY ({green jobs})	491
Q3_GJ	TITLE-ABS-KEY ({green-job})	0
Q4_GJ	TITLE-ABS-KEY ({green-jobs})	3
Q5_GJ	TITLE-ABS-KEY ({'green-job'})	0
Q6_GJ	TITLE-ABS-KEY ({'green-jobs'})	0
Q7_GJ	TITLE-ABS-KEY ({'green' job})	1
Q1_GJ	TITLE-ABS-KEY ({'green' jobs})	9
Q2_GJ	TITLE-ABS-KEY ({'green job'})	2
Q3_GJ	TITLE-ABS-KEY ({'green jobs'})	27
Q4_GJ	TLE-ABS-KEY ({green job} OR {green jobs} OR {green-jobs} OR {'green' job} OR {'green' jobs} OR {'green job'} OR {'green jobs'})	559/557♠

Source: Authors elaboration. ♠Accepted after reading for further study.

Table S2. Syntaxes used in Queries calibration for the Scopus scientific database exploration variants of naming green collar

No.	Query Syntax	No. of results (10 September 2022)
Q1_GC	TITLE-ABS-KEY ({green collar})	32/30♠
Q2_GC	TITLE-ABS-KEY ({green collars})	1/0
Q3_GC	TITLE-ABS-KEY ({green-collar})	27/26♠
Q4_GC	TITLE-ABS-KEY ({green-collars})	0
Q5_GC	TITLE-ABS-KEY ({'green-collar'})	4
Q6_GC	TITLE-ABS-KEY ({'green-collars'})	0
Q7_GC	TITLE-ABS-KEY ({'green' collar})	0
Q8_GC	TITLE-ABS-KEY ({'green' collars})	0
Q9_GC	TITLE-ABS-KEY ({'green collar'})	2
Q10_GC	TITLE-ABS-KEY ({'green collars'})	0
Q11_GC	TITLE-ABS-KEY ({green collar} OR {green-collar} OR {'green-collar'} OR {'green collar'})	57/51♠

Source: Authors elaboration. ♠Accepted after reading for further study.

Table S3. Syntaxes used in Queries calibration for the Scopus scientific database exploration variants of naming green employment

No.	Query Syntax	No. of results (10 September 2022)
Q1_GE	TITLE-ABS-KEY ({green employment})	29
Q2_GE	TITLE-ABS-KEY ({green employments})	2
Q3_GE	TITLE-ABS-KEY ({'green' employment})	0
Q4_GE	TITLE-ABS-KEY ({'green' employments})	0
Q5_GE	TITLE-ABS-KEY ({'green employment'})	0
Q6_GE	TITLE-ABS-KEY ({'green employments'})	0
Q7_GE	TITLE-ABS-KEY ({green employment} OR {green employments})	31

Source: Authors elaboration.

Table S4. Syntaxes used in Queries calibration for the Scopus scientific database exploration variants of naming environmental job

No.	Query Syntax	No. of results (10 September 2022)
Q1_EJ	TITLE-ABS-KEY ({environmental job})	20
Q2_EJ	TITLE-ABS-KEY ({environmental jobs})	15
Q3_EJ	TITLE-ABS-KEY ({'environmental' job})	0
Q4_EJ	TITLE-ABS-KEY ({'environmental' jobs})	0
Q5_EJ	TITLE-ABS-KEY ({'environmental job'})	0
Q6_EJ	TITLE-ABS-KEY ({'environmental jobs'})	1
Q7_EJ	TITLE-ABS-KEY ({environmental job} OR {environmental jobs} OR {'environmental job'})	32

Source: Authors elaboration.

Table S5. Syntaxes used in Queries calibration for the Scopus scientific database exploration variants of naming sustainability job

No.	Query Syntax	No. of results (10 September 2022)
Q1_SJ	TITLE-ABS-KEY ({sustainability job})	2
Q2_SJ	TITLE-ABS-KEY ({sustainability jobs})	0
Q3_SJ	TITLE-ABS-KEY ({'sustainability' job})	0
Q4_SJ	TITLE-ABS-KEY ({'sustainability' jobs})	0
Q5_SJ	TITLE-ABS-KEY ({'sustainability job'})	0
Q6_SJ	TITLE-ABS-KEY ({'sustainability jobs'})	0

Source: Authors elaboration.
